# The Complete Sequence and Comparative Analysis of a Multidrug-Resistance and Virulence Multireplicon IncFII Plasmid pEC302/04 from an Extraintestinal Pathogenic *Escherichia coli* EC302/04 Indicate Extensive Diversity of IncFII Plasmids

**DOI:** 10.3389/fmicb.2015.01547

**Published:** 2016-01-11

**Authors:** Wing Sze Ho, Kien-Pong Yap, Chew Chieng Yeo, Ganeswrie Rajasekaram, Kwai Lin Thong

**Affiliations:** ^1^Faculty of Science, Institute of Biological Sciences, University of MalayaKuala Lumpur, Malaysia; ^2^Faculty of Medicine, Biomedical Research Centre, Universiti Sultan Zainal AbidinKuala Terengganu, Malaysia; ^3^Department of Pathology, Hospital Sultanah Aminah Johor BharuJohor Bharu, Malaysia

**Keywords:** comparative genomics, extraintestinal pathogenic *E. coli*, ExPEC, hybrid plasmid, multireplicon IncFIIA plasmids, multidrug-resistance, transmissible, virulence

## Abstract

Extraintestinal pathogenic *Escherichia coli* (ExPEC) that causes extraintestinal infections often harbor plasmids encoding fitness traits such as resistance and virulence determinants that are of clinical importance. We determined the complete nucleotide sequence of plasmid pEC302/04 from a multidrug-resistant *E. coli* EC302/04 which was isolated from the tracheal aspirate of a patient in Malaysia. In addition, we also performed comparative sequence analyses of 18 related IncFIIA plasmids to determine the phylogenetic relationship and diversity of these plasmids. The 140,232 bp pEC302/04 is a multireplicon plasmid that bears three replication systems (FII, FIA, and FIB) with subtype of F2:A1:B1. The plasmid is self-transmissible with a complete transfer region. pEC302/04 also carries antibiotic resistance genes such as *bla*_TEM−1_ and a class I integron containing *sul1, cml* and *aadA* resistance genes, conferring multidrug resistance (MDR) to its host, *E. coli* EC302/04. Besides, two iron acquisition systems (SitABCD and IutA-IucABCD) which are the conserved virulence determinants of ExPEC-colicin V or B and M (ColV/ColBM)-producing plasmids were identified in pEC302/04. Multiple toxin-antitoxin (TA)-based addiction systems (i.e., PemI/PemK, VagC/VagD, CcdA/CcdB, and Hok/Sok) and a plasmid partitioning system, ParAB, and PsiAB, which are important for plasmid maintenance were also found. Comparative plasmid analysis revealed only one conserved gene, the *repA1* as the core genome, showing that there is an extensive diversity among the IncFIIA plasmids. The phylogenetic relationship of 18 IncF plasmids based on the core regions revealed that ColV/ColBM-plasmids and non-ColV/ColBM plasmids were separated into two distinct groups. These plasmids, which carry highly diverse genetic contents, are also mosaic in nature. The atypical combination of genetic materials, i.e., the MDR- and ColV/ColBM-plasmid-virulence encoding regions in a single ExPEC plasmid is rare but of clinical importance. Such phenomenon is bothersome when the plasmids are transmissible, facilitating the spread of virulence and resistance plasmids among pathogenic bacteria. Notably, certain TA systems are more commonly found in particular ExPEC plasmid types, indicating the possible relationships between certain TA systems and ExPEC pathogenesis.

## Introduction

*Escherichia coli* is a highly versatile bacterium and often exists as a commensal in the gastrointestinal tract of humans and animals (Russo and Johnson, 2000). However, it can also colonize, infect, and cause diseases at both intra- and extraintestinal sites. The extraintestinal pathogenic *E. coli* (ExPEC), a major subgroup of *E. coli*, can infect almost any possible extraintestinal sites, in both normal and immuno-compromised hosts (Russo and Johnson, 2000). Certain virulence determinants such as iron acquisition systems which are essential for the extraintestinal adaptation of ExPECs can also be located in chromosomes and/or mobile genetic elements like plasmids (Sabri et al., 2006; Johnson et al., 2010a).

A bacterial plasmid is an important agent for introducing adaptive traits horizontally to bacterial hosts as well as contributing to bacterial pathogenesis and evolution. Plasmids that carry virulence and resistance genes besides genes essential for their own transmission and maintenance are termed virulence plasmids and resistance plasmids, respectively (Sengupta and Austin, 2011). Large virulence plasmids (>100 kb) belonging to the incompatibility group IncF are widely disseminated in clinically relevant *Enterobacteriaceae* (Villa et al., 2010) including extraintestinal pathogenic *E. coli* (Johnson and Nolan, 2009; Mellata et al., 2009; Peigne et al., 2009; Woodford et al., 2009; Johnson et al., 2010a; Brolund et al., 2013). An example of an IncF virulence plasmid that is often associated with ExPEC is the colicin-producing plasmid. Colicins are antimicrobial substances produced by certain members of colicin-producing *Enterobacteriaceae* that can kill susceptible strains, contributing to the virulence of the bacterial host (Waters and Crosa, 1991). Colicin V (ColV) and colicin B and M (ColBM) are among the most common types of colicins described that are associated with IncF plasmids. These plasmids are also known to harbor a repertoire of virulence determinants (extensively reviewed by Johnson and Nolan, 2009) that are essential for ExPEC pathogenesis. Nonetheless, putative evolutionary intermediates of ColV/ColBM plasmids which harbor the incomplete classical ColV components have also been reported, albeit rarely (Johnson and Nolan, 2009). Notoriously, IncF plasmids are also responsible for the dissemination of CTX-M genes that confer resistance to the first-line antimicrobial therapy against *Enterobacteriaceae* (i.e., cephalosporins; Villa et al., 2010; Cantón et al., 2012; Brolund et al., 2013). In fact, the successful global dissemination of IncF plasmids may be attributed to its multi-replicon characteristic which enable replication in a broader range of hosts (Villa et al., 2010).

Here, we characterized the plasmid sequence (pEC302/04) of a multidrug-resistant ExPEC (EC302/04) that was recovered from the tracheal aspirate of a patient that was admitted to an intensive care unit in Malaysia. We also performed an extensive comparative plasmid analysis of the pEC302/04 plasmid with an additional 17 IncFIIA plasmids that were obtained from the public database. The outcome of our study provides new insights into the diversity and phylogeny of multireplicon IncFIIA plasmids, particularly in regards to hybrid plasmids that carry both resistance and virulence determinants.

## Materials and methods

### Bacterial strain and plasmid

*E. coli* strain EC302/04 is a multidrug-resistant (MDR) strain that was isolated from the tracheal aspirate of a patient admitted to an intensive care unit in a Malaysian tertiary hospital in the year 2004 (Ho et al., 2012a). EC302/04 is regarded as an ExPEC based on the isolation site as well as the presence of ExPEC-associated virulence determinants such as iron acquisition systems and adhesins (Köhler and Dobrindt, 2011; Pitout, 2012). The ExPEC EC302/04 harbors a plasmid (pEC302/04) and also belongs to the uncommon sequence type ST349 and serotype O166:H15 (Ho et al., 2012a).

### Plasmid gap closing and annotation

The draft plasmid genome of pEC302/04 was assembled as part of the EC302/04 next-generation whole genome sequencing (WGS) study (Ho et al., 2012a). Contigs putatively derived from pEC302/04 were determined by subjecting the whole genome sequence of strain EC302/04 to BLASTn against the non-redundant nucleotide collection in GenBank. Two plasmid sequences of *E. coli* (pEC_L46, accession no. GU371929.1; pEC_L8, accession no. GU371928.1) and a genome sequence of *Salmonella enterica* serovar Typhimurium (T000240, accession no. AP011957.1) that showed the highest coverage and nucleotide similarities with pEC302/04 contig sequences were used as the reference sequences for plasmid gap closing. Bacterial genomes finishing tool (Galardini et al., 2011) was used for mapping the contigs against the reference genomes and primers were designed for PCR gap closing (Supplementary Table 1). Amplified products were purified and submitted to a commercial facility for conventional Sanger sequencing. Plasmid sequence was submitted to the RAST server for annotation (Aziz et al., 2008). Virulence genes were identified by mapping the annotated sequences against the Virulence Factor Database (VFDB) (Chen et al., 2012) using BLASTn where BLAST hits with *e*-value ≤ 1e–10, query coverage ≥80% and nucleotide identity ≥80% were considered as positive hits. Insertion-sequence (IS) elements were identified by tBLASTx searches against IS-finder (https://www-is.biotoul.fr/). Toxin-antitoxin systems were identified using TAfinder using default parameters (http://202.120.12.133/TAfinder/index.php) and the identified toxins and antitoxins were further validated using BLASTp. All annotations were then manually curated.

### Conjugation and confirmation of transconjugants

The transmissibility of plasmid pEC302/04 was determined using liquid conjugation experiment as described previously (Johnson et al., 2010a) at 37°C with two biological replicates. EC302/04 and nalidixic-acid resistant *E. coli* DH5α were used as donor and recipient cells, respectively. *E. coli* DH5α (pEC302/04) transconjugants were confirmed by: (a) selective plating on Luria-Bertani (LB) agar plates containing a donor-inhibiting concentration of ampicillin (100 μg) (Sigma-Aldrich) and recipient-inhibiting concentration of nalidixic acid (30 μg/ml); (b) PCR detection of plasmid-encoded *bla*_TEM−1_ gene which was present only in the donor strain EC302/04 using a previously described primer pair (Oliver et al., 2002); and (c) PFGE profiling of the donor, recipient cell and transconjugants which were performed according to Ho et al. (2012b). The conjugation frequency (number of transconjugants per donor cell) was calculated using the averages of two biological and three technical replicates.

### Antimicrobial susceptibility testing and identification of resistance genes

Resistance phenotypes for EC302/04, DH5α (pEC302/04) and *E. coli* DH5α were determined using the disk diffusion method according to Clinical and Laboratory Standards Institute (CLSI) guidelines (2015) CLSI (2015) on Mueller-Hinton agar (BD). The antimicrobial agents in commercial disks (Oxoid Ltd.) used were: ampicillin (AMP, 10 μg), tetracycline (TET, 30 μg), trimethoprim/sulfamethoxazole (SXT, 30 μg), nalidixic acid (NAL, 30 μg), ciprofloxacin (CIP, 5 μg), streptomycin (STR, 10 μg), spectinomycin (SPC, 100 μg), compound sulfonamides (S300, 300 μg), kanamycin (KAN, 30 μg), chloramphenicol (CHL, 30 μg), amoxicillin/clavulanic acid (AMC, 20/10 μg), ceftriaxone (CRO, 30 μg), cefotaxime (CTX, 30 μg), aztreonam (ATM, 30 μg), cefepime (FEP, 30 μg), cefoperazone (CFP, 30 μg), ceftazidime (CAZ, 30 μg), gentamicin (GEN, 10 μg), tobramycin (10 μg), amikacin (AMK, 30 μg), meropenem (MEM, 10 μg), and imipenem (IPM, 10 μg). *E. coli* ATCC 25922 was used as control strain (CLSI, 2015).

The identification of acquired resistance genes for the plasmid pEC302/04 was performed by submitting the complete plasmid nucleotide sequence to ResFinder web server (https://cge.cbs.dtu.dk//services/ResFinder/) (Zankari et al., 2012).

### Bacterial growth assay in iron-limited media and analysis

The donor *E. coli* EC302/04, DH5α (pEC302/04) transconjugant and recipient DH5α were grown in LB broth overnight (16–18 h) at 37°C. Cultures were diluted to OD_600nm_ of 0.05–0.1 into LB broth and LB broth supplemented with iron chelator 2'2-dipyridyl (DIP) (Sigma-Aldrich, Steinheim, Germany) (at concentrations of 100, 200, and 300 μM). The growth assay was performed in 96 well plates at 37°C with readings at 600 nm taken every 15 min for 24 h using a Spectramax spectrophotometer (Molecular Devices, USA). Growth kinetics for all 3 strains in each type of the media was determined using the average values of three independent biological and three technical replicates. The growth rates of each replicate for all three strains in different media were determined using GrowthRates (Hall et al., 2014).

### Presence of iron acquisition systems in *E. coli* DH5α and EC302/04

The nucleotide sequences of all four types of commonly found iron acquisition systems in *E. coli* that are listed in VFDB (i.e., aerobactin, enterobactin, Chu system, and IroN) were retrieved from http://www.mgc.ac.cn/VFs/ (Chen et al., 2012; Supplementary Table 2). The presence of these iron uptake systems in the *E. coli* DH5α (accession no.: JRYM00000000.1) and EC302/04 (accession no.: AMFM00000000.1) genomes were determined using BLASTn (*E*-value = 0, query coverage ≥95%, identity ≥95%).

### Comparative plasmid sequence and phylogenetic analysis

To better understand the diversity and evolution of IncFIIA plasmids, a total of 17 fully sequenced IncFIIA plasmids were retrieved from GenBank (Table 1). These plasmids were selected based on the closest homology to pEC302/04 using BLASTn (*E*-value = 0, query coverage ≥ 30%, identity ≥ 98%; Altschul et al., 1997). The genes of all 18 IncF plasmids were reannotated using RAST (Aziz et al., 2008). The homologous genes of 18 IncFIIA plasmids were determined using PGAP (Zhao et al., 2012) and Hal pipeline (Robbertse et al., 2011).

**Table 1:** **Information of the 18 IncF plasmids that are used in the comparative plasmid sequence analysis**.

**Plasmid**	**Strain origin**	**Size (bp)**	**Acession number**	**Remark**
pEC302/04	ExPEC	140232	CP011493	Non-ColV/ColBM
pEC_L46	*E. coli*	144871	GU371929.1	Non-ColV/ColBM
pEC_L8	*E. coli*	118525	GU371928.1	Non-ColV/ColBM
pEK499	*E. coli*	117536	EU935739.1	Non-ColV/ColBM
pEFC36a	uncultured bacterium	87419	JX486126.1	Non-ColV/ColBM
pRSB225	uncultured bacterium	164550	JX127248.1	Non-ColV/ColBM
pRSB107	uncultured bacterium	120592	AJ851089.1	Non-ColV/ColBM
pSH163_120	*S. enterica* Heidelberg	120524	JN983046	Non-ColV/ColBM
pSH696_117	*S. enterica* Heidelberg	117278	JN983047	Non-ColV/ColBM
pAPEC-1	ExPEC/APEC	103275	CP000836	ColV/ColBM
pAPEC-O1-ColBM	ExPEC/APEC	174241	DQ381420	ColV/ColBM
pAPEC-O103-ColBM	ExPEC/APEC	124705	CP001232	ColV/ColBM
pAPEC-O2-ColV	ExPEC/APEC	184501	AY545598	ColV/ColBM
pCS0010A	*S. enterica* Kentucky	146811	CP002090	ColV/ColBM
pCVM29188_146	*S. enterica* Kentucky	146811	CP001122	ColV/ColBM
pECOS88	ExPEC/NMEC	133853	CU928146	ColV/ColBM
pO83_CORR	AIEC	147060	CP001856	ColV/ColBM
pS286ColV	ExPEC/NMEC	97818	HF922624.1	ColV/ColBM

The core regions of the 18 IncFIIA plasmids (including the intergenic regions) were determined using Reference sequence Alignment-based Phylogeny (REALPHY) builder (Bertels et al., 2014). Nucleotide sequences of the *repA1* and *tra* genes (*traI, traJ, traM, traY, traT, and traS*) were extracted from GenBank and aligned using MAFFT (Katoh et al., 2002) with default parameters. FindModel (Posada and Crandall, 2001) was then used to determine the best-fit phylogenetic model for each of the nucleotide sequence alignment. The phylogenetic trees (based on core regions, *repA1* and 6 *tra* nucleotide sequences) of the 18 plasmids were then constructed with MEGA 5 (Tamura et al., 2011) using maximum-likelihood (ML) method (General Time Reversible plus Gamma model) with 1000 pseudo-replicates. To facilitate visualization on the extensive diversity of the 18 studied IncFIIA plasmids, circular maps displaying the core and accessory genomes of all the studied plasmids were visualized using Gview Server (Petkau et al., 2010), with plasmid pEC302/04 assigned as the seed genome. TA systems for all plasmids genomes were identified as described in Section Plasmid Gap Closing and Annotation.

The multilocus sequence types of the 18 IncF plasmids were determined *in silico* using the PubMLST server (http://pubmlst.org/perl/bigsdb/bigsdb.pl?db=pubmlst_plasmid_seqdef&page=sequenceQuery). Due to the multi-replicon nature of IncF plasmids, the FAB formula (Villa et al., 2010) was applied to assign allelic profiles for three types of IncF replicons in the 18 plasmids, where “F” represents IncFII replicon, “A” represents FIA and “B” represents FIB. For instance, if a plasmid X has an FAB formula of F1:A2:B-, it indicates that plasmid X carries an FII replicon with assigned allelic profile of “1,” a FIA replicon with allelic profile “2”; while the FIB replicon is absent.

We have included 11 diverse representative plasmids (including pEC302/04) from various sources (such as waste water treatment plant, human urinary tract, avian samples, etc.) for further detailed comparative plasmid sequence analysis using Easyfig (Sullivan et al., 2011). Another simplified comparative plasmid map supplemented with GC content was also illustrated by including genetically highly similar plasmids, as well as IncFIIA plasmids that were not included in the earlier detailed plasmid map.

### Nucleotide sequence accession number

The annotated plasmid sequence of pEC302/04 was deposited in the GenBank database under accession number CP011493.

## Results and discussion

### Analysis of plasmid pEC302/04

**Figure 1:**
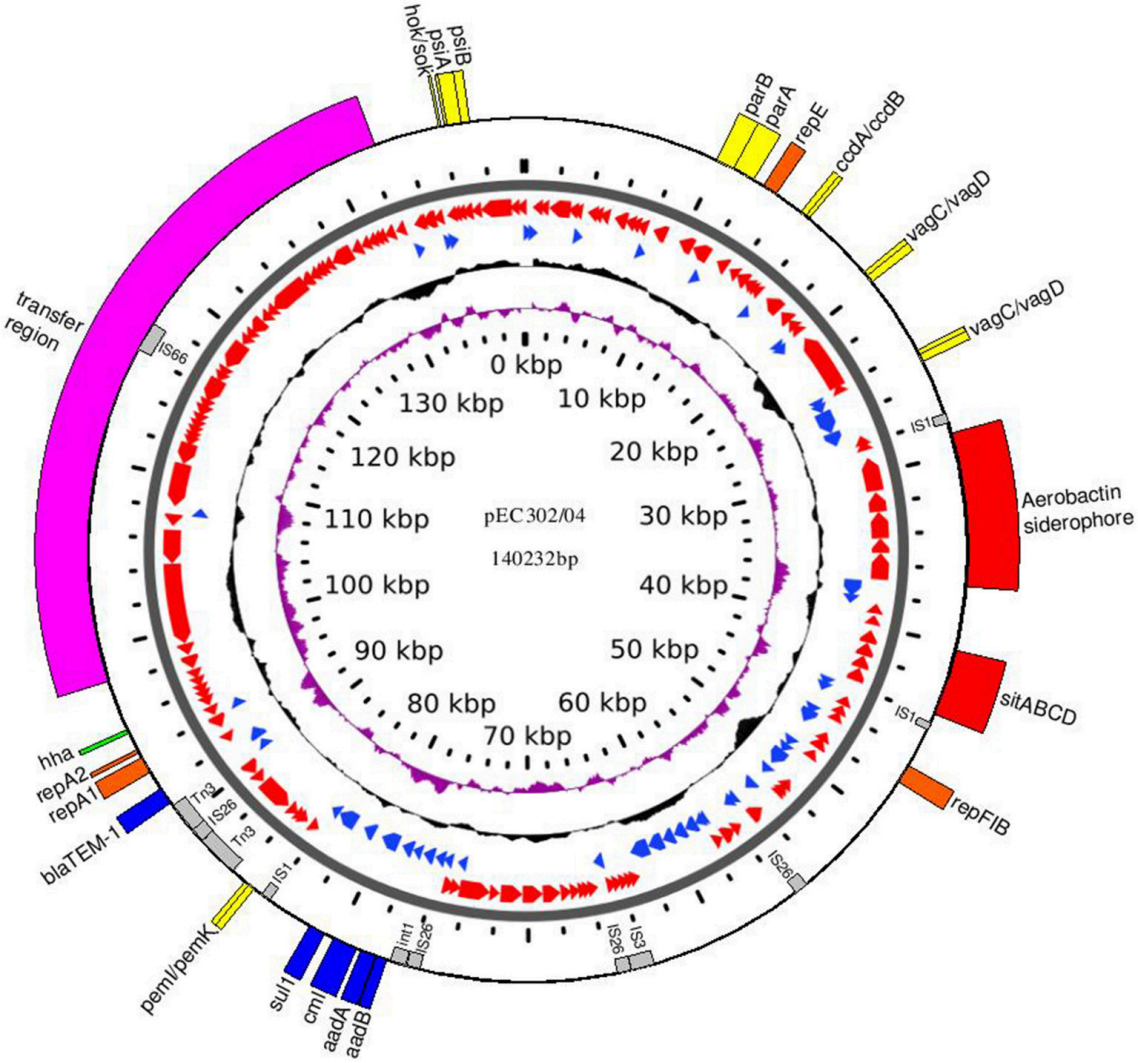
**Circular map of plasmid pEC302/04**. The 1st (innermost ring) and the 6th rings show the plasmid size in kbp. The 2nd and 3rd rings (from inner to outer) show the GC skew (purple) and GC content (black), respectively. The 4th and 5th rings show the ORFs in forward (blue) and reverse orientation (red). The outermost ring shows the important genes encoding regions highlighting with different colors according to its function: orange, replication; pink, transfer region; yellow, addictive systems; red, iron acquisition systems; blue, antimicrobial resistance; green, other virulence factor; gray, IS elements.

pEC302/04 is a circular multi-replicon IncFIIA plasmid (140,232 bp) with an average GC content of 52% (Figure 1). A total of 184 ORFs were predicted and annotated using RAST (Aziz et al., 2008) which were then manually curated to improve the annotations (Supplementary Table 3). A total of three replicons were found for pEC302/04, namely RepFIA, RepFIIA, and RepFIB with the multireplicon F plasmid FAB formula of F2:A1:B1.

Different plasmid maintenance systems are required to achieve stable plasmid inheritance in the bacterial host and these include plasmid segregation and addiction systems (Sengupta and Austin, 2011). The *parABS* genes, which are responsible for an active plasmid partitioning system, were found upstream of the *tra* region of pEC302/04. The partition system is important in ensuring each daughter cell receives a plasmid following cell division (Kroll et al., 2010). Besides, several toxin-antitoxin (TA)-based addiction systems were also found on pEC302/04: (i) PemI-PemK TA system (Pem for plasmid emergency maintenance) is found associated with a transfer inhibition protein (Tir); (ii) CcdA-CcdB TA system (Ccd for coupled cell division) is found near to the FIA replicon; (iii) two copies of VagC-VagD TA systems (Vag for virulence-associated gene) are separated by 2 ORFs encoding hypothetical proteins and are found near to another addiction system, CcdA-CcdB and (iv) Hok-Sok TA system (Hok for host killing and Sok for suppression of killing) is found next to the gene that codes for PsiA-PsiB (plasmid SOS inhibition protein; Figure 1). The presence of multiple plasmid maintenance systems in pEC302/04 is likely to ensure stable plasmid inheritance particularly in the absence of selective pressure.

**Figure 2:**
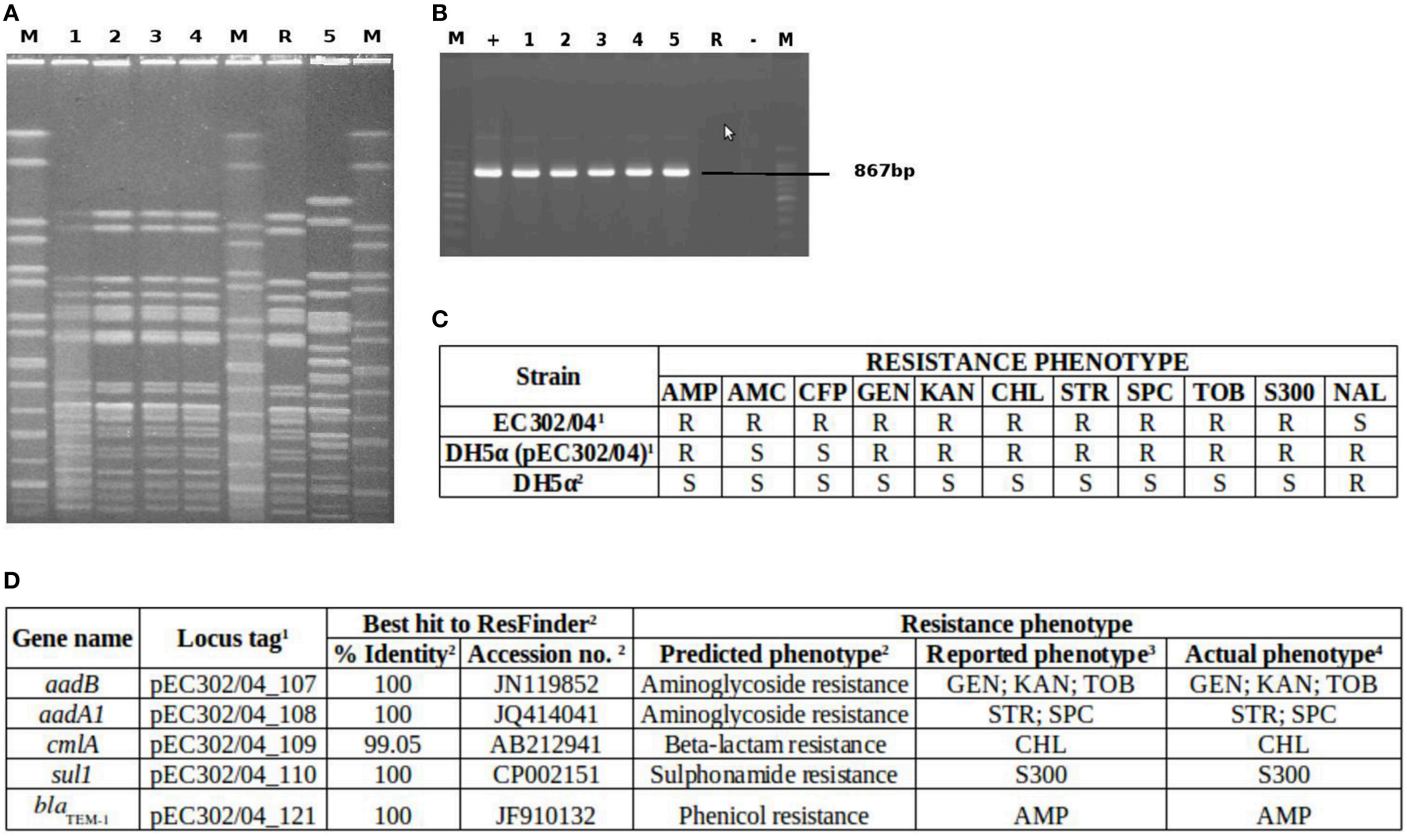
**Confirmation of pEC302/04 transfer and resistance phenotypes and genotypes of EC302/04, *E. coli* DH5α and DH5α (pEC302/04) transconjugant**. **(A)** PFGE gel of DH5α (pEC302/04) transconjugants, *E. coli* DH5α and EC302/04. Lane M, *XbaI* digested H9812 DNA marker; Lanes 1–4, DH5α (pEC302/04); Lane R, *E. coli* DH5α; Lane 5, EC302/04. **(B)** *bla*_TEM_ detection in transconjugants DH5α (pEC302/04), DH5α and EC302/04. Lane M, 100bp ladder; Lane +, positive control strain EC1003-1; Lane 1–4, DH5α (pEC302/04); Lane 5, EC302/04; Lane R, *E. coli* DH5α; Lane −, no-template (negative) control **(C)** Resistance phenotypes of transconjugants DH5α (pEC302/04), *E. coli* DH5α and EC302/04. ^1^strain harboring plasmid pEC302/04; ^2^strain without plasmid pEC302/04; R, non-susceptible (including resistant and intermediate resistant); S, sensitive **(D)**. In silico analysis of pEC302/04-encoded resistance genes.^1^refer to Supplementary Table 3. ^2^*In silico* results obtained from ResFinder. ^3^Resistance phenotypes reported by other studies. ^4^Resistance phenotypes determined using antimicrobial susceptibility testing according to CLSI (CLSI, 2015).

The complete *tra* region which codes for the transfer components of plasmids was also identified in pEC302/04 and it spans approximately a quarter (36 kb) of the plasmid. This *tra* region harbors 24 *tra* genes (*traM, traJ, traY, traA, traL, traE, traK, traB, traP, traV, traR, traC, traW, traU, traN, traF, traQ, traH, traG, traS, traT, traD, traI*, and *traX*), 9 *trb* genes (*trbD, trbG, trbI, trbC, trbE, trbA, trbB, trbJ*, and *trbF*) and the regulatory fertility inhibition gene (*finO*) which act as a conjugal transfer repressor (Yoshioka et al., 1987). To investigate if pEC302/04 is self-transmissible, conjugation experiments were carried out for *E. coli* strain EC302/04 using *E. coli* DH5α as the recipient. Transconjugants were detected at a frequency of 5 × 10^−4^. Subsequent PCR detection of the *bla*_TEM−1_ gene carried on pEC302/04 in the transconjugants, and PFGE of the donor, recipient and transconjugant cells (Figure 2) have demonstrated that pEC302/04 was indeed self-transmissible.

Comparison of the resistance phenotypes of EC302/04, *E. coli* DH5α and transconjugant DH5α (pEC302/04) revealed that genes conferring resistance to ampicillin, kanamycin, streptomycin, spectinomycin, compound sulfonamides, gentamicin, tobramycin, and chloramphenicol have been transferred to the DH5α (pEC302/04) transconjugant (Figure 2C). *In silico* analysis of resistance genes found in the pEC302/04 sequence also showed concordant findings with the antimicrobial resistance phenotypes of the transconjugants. A class I integron harboring the *aadA1* gene (conferring resistance to streptomycin and spectinomycin), *aadB* (resistance to gentamicin, kanamycin, and tobramycin) and *cmlA* (non-enzymatic chloramphenicol resistance gene that encodes for an efflux pump) were identified in pEC302/04. Besides the resistance determinants, *sulI* (resistance to sulfonamides) and *qacE*Δ (quatenary ammonium compound-resistance gene) which are the 3′-conserved segment of class I integrons (Mazel, 2006) were also found in the above-mentioned integron. Notably, pEC302/04 carries a Tn*3* transposon bearing the *bla*_TEM−1_ (ampicillin resistance) (Figure 1). Collectively, the findings revealed that the non-susceptibility to amoxicillin/clavulanic acid and cefoperazone, which are often associated with *bla*_AmpC_ (Cantón et al., 2008), is not plasmid pEC302/04-mediated in EC302/04.

Twenty-four putative virulence factors (VFs) were identified in pEC302/04 with majority (*n* = 9) of the VFs being associated with iron acquisition systems. The region encoding two iron acquisition systems constitutes the putative virulence region which spans approximately 16 kb of the plasmid pEC302/04. The aerobactin siderophore-encoding gene clusters (*iutA-iucABC*D) were found adjacent to *sitABCD* (encoding for iron and manganese transport system) and separated by genes encoding ShiF (putative membrane transport protein) and Eno (phosphopyruvate hydratase). Both systems are reportedly required for growth under iron depleted condition (Boyer et al., 2002) as well as increased virulence both *in vivo* and *in vitro* (Boyer et al., 2002; Sabri et al., 2006). Besides iron acquisition systems, virulence determinants with other functions were also identified. The *traJ* gene, which codes for a plasmid conjugation transfer protein that may play an important role in invasion (Hill et al., 2004), was found in pEC302/04. We also identified a complete transfer region which encodes for proteins corresponding to type IV pilus (Lawley et al., 2003; Zahrl et al., 2006), which has been described as an urovirulence determinants for ExPEC (UPEC in particular; Kulkarni et al., 2009).

Transposable elements are often associated with genes encoding fitness traits such as resistance and virulence genes (de Lorenzo et al., 1988; Szczepanowski et al., 2005; Fricke et al., 2008; Kiiru et al., 2013). Ten ORFs belonging to insertion sequences and transposons of five main families (IS*1*, IS*3*, IS*6*, IS*66*, and Tn*3*) was found in pEC302/04. Genes encoding the two putative iron acquisition systems (*iuc/iutABCD* and *sitABCD*) were collectively flanked by two copies of IS*1* in an inverted orientation, a genetic organization which resembled a composite transposon (Figure 1). Although, the IS*1*-flanked aerobactin siderophore encoding DNA fragment has been demonstrated experimentally to be transposable in *E. coli* (Perez-casal and Crosa, 1984; de Lorenzo et al., 1988), the target site-duplication (evidence of tranposition event) was not found in pEC302/04, indicating that this IS*1*-flanked fragment may not have transposed recently. Besides virulence genes, transposable elements are also often associated with resistance genes (Kiiru et al., 2013). The *bla*_TEM−1_ resistance gene was found within the Tn*3* transposon, in which the transposase was disrupted by a copy of IS*26.* The integrase gene of the class I integron (detailed above) was also punctuated by a copy of IS*26.* These findings suggest that the integrase and transposase are likely non-functional, rendering the mobility of the class I integron and Tn*3* transposon. Interestingly, the complete transfer regions of pEC302/04 were interrupted by an insertional element, where a copy of IS*66* was inserted into the intergenic region between *traN* and *trbE* genes, without affecting the self-transmissibility of pEC302/04.

### Growth in iron-limited medium and the presence of iron acquisition systems in *E. coli* DH5α and EC302/04

**Figure 3:**
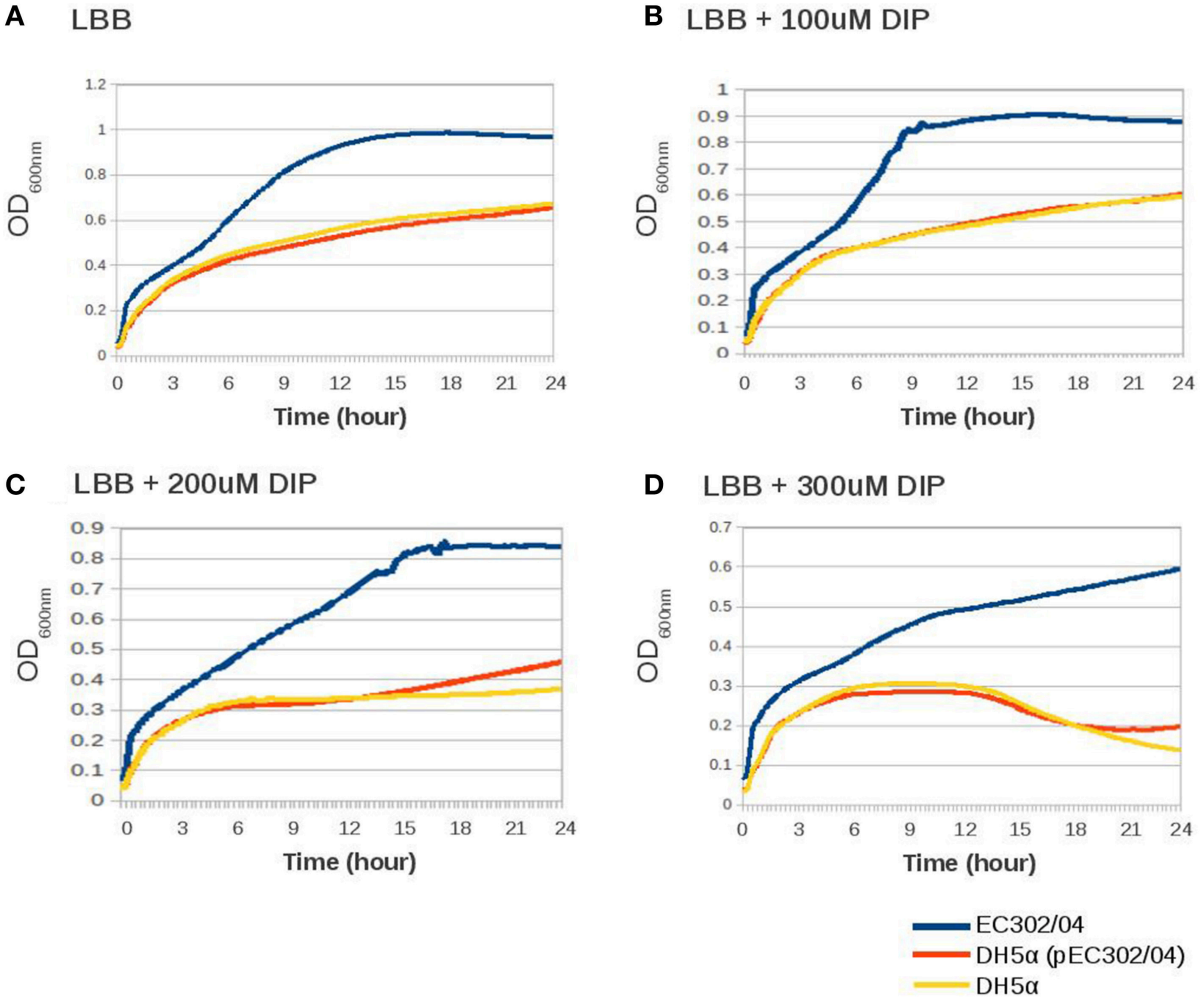
**Growth curves of EC302/04, *E. coli* DH5α and DH5α (pEC302/04) transconjugant in rich and iron-limited medium**. **(A)** LB broth; **(B)** LB broth supplemented with 100 μM of DIP; **(C)** LB broth supplemented with 200 μM of DIP; **(D)** LB broth supplemented with 300 μM of DIP.

Iron, which is essential for bacterial growth, is present at very low concentration at the extraintestinal sites (Hagan et al., 2010). The presence of two potential iron acquisition systems, i.e., the aerobactin siderophore-encoding gene cluster (*iutA-iucABCD*) and the iron/manganese transport-encoding genes (*sitABCD*) in pEC302/04 prompted us to evaluate the ability of EC302/04, *E. coli* DH5α and the DH5α (pEC302/04) transconjugant to grow in an iron-depleted medium. When all three strains were grown in LB broth supplemented with an iron chelator (DIP) at different concentrations, their respective growth were attenuated and the OD_600nm_ readings of all strains were reduced as the concentration of DIP increased (Figure 3). Nevertheless, there was no difference in growth rates between DH5α (pEC302/04) vs. *E. coli* DH5α in all rich and iron-limited media tested (Figure 3). These results indicated that the carriage of pEC302/04 for the DH5α (pEC302/04) transconjugant had no notable effect on growth in iron-limited media *in vitro*. Notably, the maximum OD reading for EC302/04 was 50–200% higher compared to that of *E. coli* DH5α and the DH5α (pEC302/04) transconjugant across all media tested (Figure 3), suggesting that EC302/04 might harbor other systems which could provide better host adaptation in an iron-depleted environment. To support this hypothesis, different types of iron acquisition systems in the genomes of *E. coli* DH5α and EC302/04 were determined. Indeed, the EC302/04 was found to harbor two additional iron acquisition systems, namely the enterobactin and Chu-type iron uptake systems. On the other hand, *E. coli* DH5α only contained genes for the enterobactin iron acquisition system (Supplementary Table 2). The redundancy of iron acquisition systems in the EC302/04 suggests that this strain may have better adaptability in iron-limited media when compared to *E. coli* DH5α, hence its better growth rate in media supplemented with DIP.

### Multireplicon IncFIIA plasmids displayed high genetic diversity and mosaicism with single *repA1* gene as the core region

**Figure 4:**
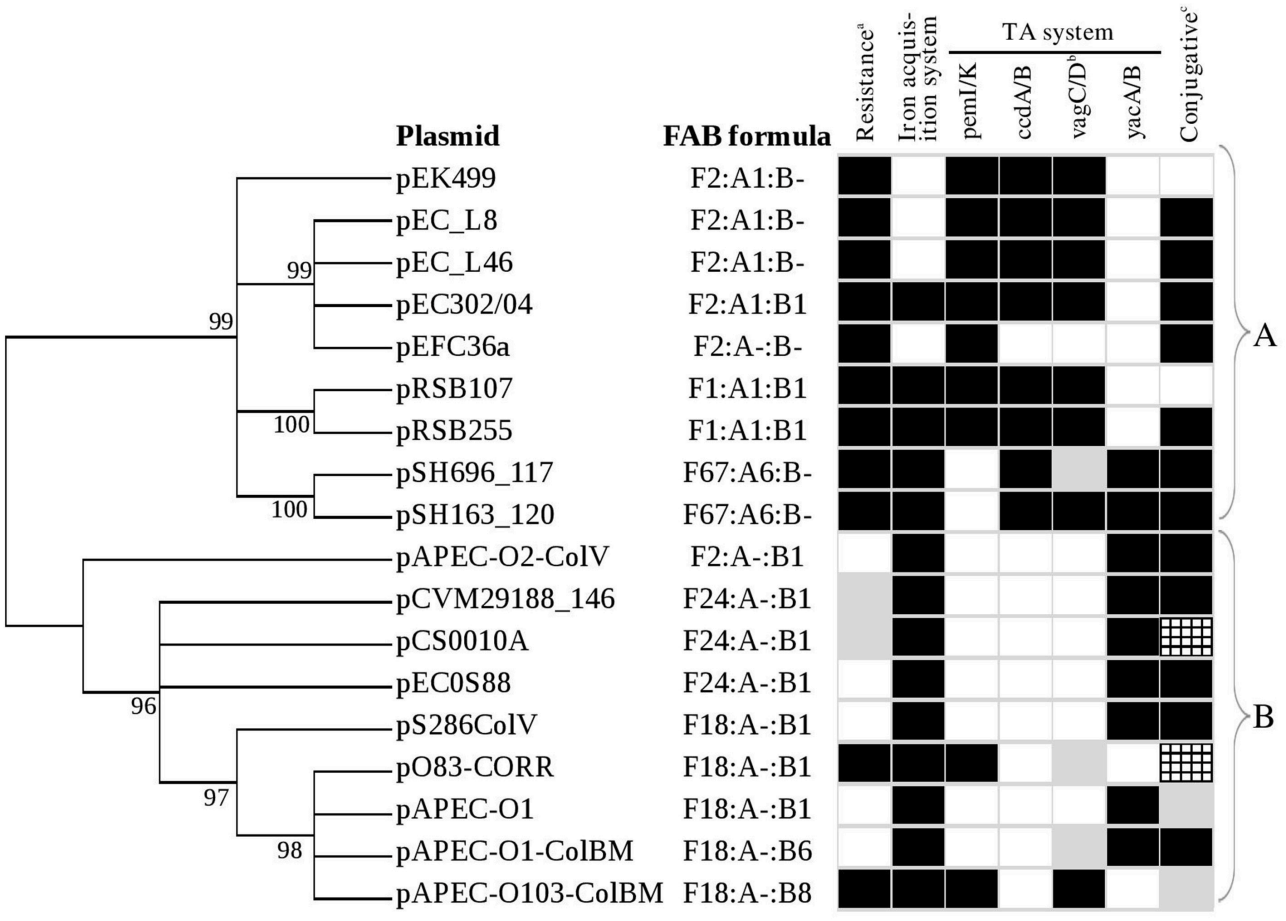
**Phylogenetic tree of RepFII *repA1* gene from 18 IncFIIA plasmids**. Core genome determination using both PGAP and Hal phylogenomics revealed that *repA1* is the only gene conserved in all 18 plasmids. Trees constructed using output of PGAP and Hal phylogenomics are similar. Maximum likelihood (ML) method under the General Time Reversible plus Gamma model was used to construct the phylogenetic tree using MEGA5 supported with bootstrapping (1000 replicates). Bootstrap value with percentages equals or greater than 50% were shown on the branches and value less than 50% have been collapsed. FAB formula (F-:A-:B-) are indicated. All non ColV/BM plasmids were grouped together at cluster A and while all ColV/BM plasmids were grouped at cluster B. Black and white boxes indicate the presence and absence of specific plasmid features. Gray boxes^ab^ indicate the presence of specific plasmid features but are located at atypical plasmid sites. ^c^The ability to transfer for each plasmid was retrieved from their respective studies. Gray boxes^c^ indicate that the ability of the plasmid to transfer has not been reported before. Checkered gray boxes^c^ indicate that the plasmid is only transmissible in the presence of other plasmid and is not self-transmissible.

Plasmid pEC302/04 is a multireplicon plasmid with FAB formula of F2:A1:B1. IncF plasmids are known to have narrow host-range which is usually limited to *Enterobacteriaceae* (Villa et al., 2010). However, the multireplicon nature which is often observed in some IncF plasmids, including plasmid pEC302/04 (Figure 1), may allow these plasmids to adopt a broader host range (Villa et al., 2010). All plasmids used in the comparative analysis are also multi-replicon IncFIIA plasmids (except for the pECF36 with FAB formula of F2:A-:B-), reflecting the predominance of multi-replicon IncFIIA plasmids among *E. coli* isolates. The FAB formula of all 18 plasmids are also included in Figure 4.

**Figure 5:**
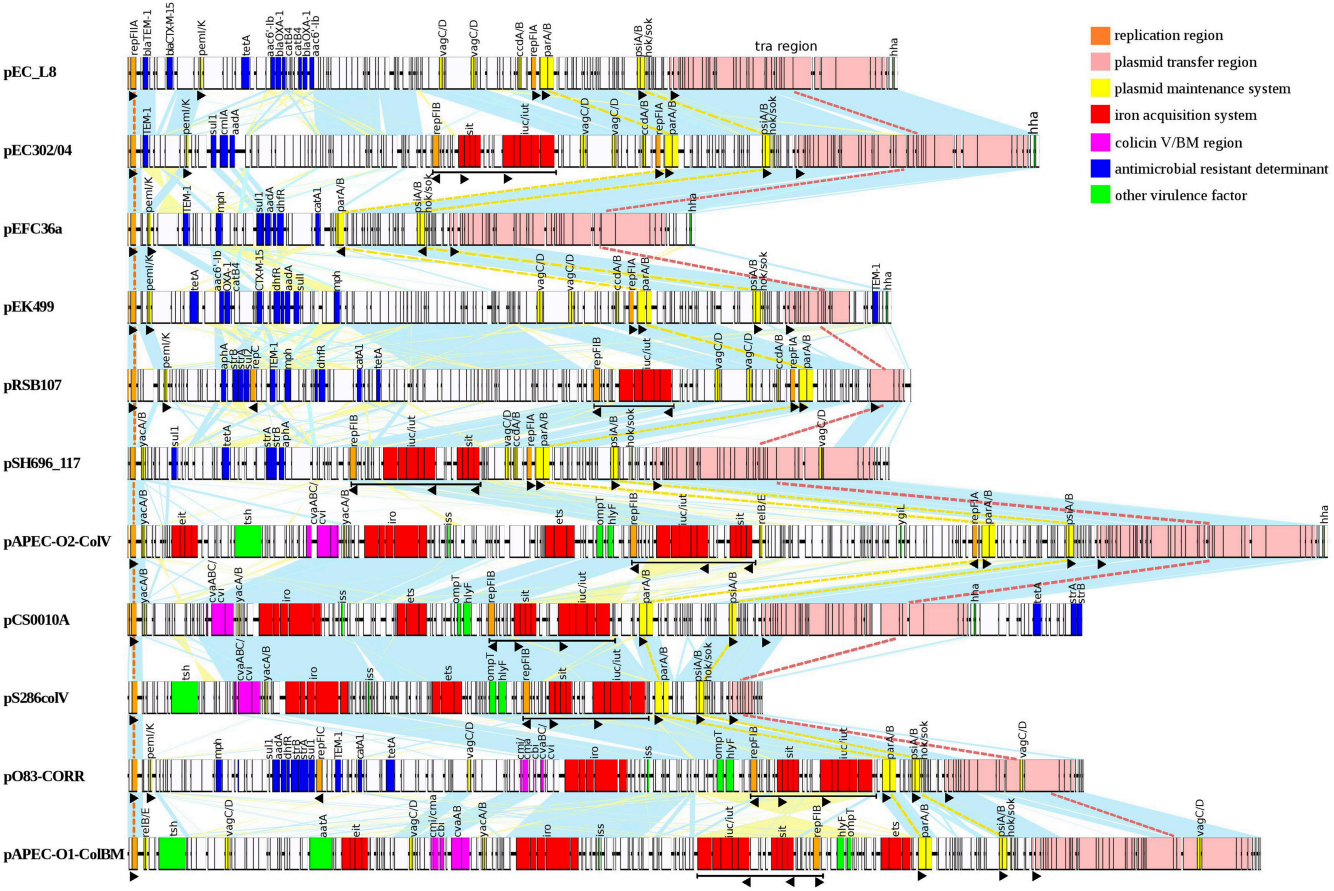
**Pairwise nucleotide comparative map of 11 multireplicon IncFIIA plasmids which are closely related to pEC302/04**. Same-strand DNA similarity is shaded light blue while reverse similarity is shaded light yellow. Coding sequences are displayed as rectangles. Major features are displayed in colors based on functional annotations: orange, replication; pink, plasmid transfer region; yellow, addictive systems; red, iron acquisition systems; purple, colicin V/BM; blue, antimicrobial resistance; green, other virulence factors; white, other genes. Black triangle depicts the orientation of specific ORF(s).

Comparative sequence analysis showed that the 18 multireplicon IncFIIA plasmids are highly diverse with only one shared gene (replication gene *repA1* of RepFIIA; Figure 5; Supplementary Figure 1), while the remaining pEC302/04 regions constitute the accessory genomes (Supplementary Figure 1). Nevertheless, most of the plasmids (except for pRSB107 and pAPEC-0103-ColBM) also harbor *parAB* and *psiAB* as the backbone (Figure 5). Both pRSB107 and pAPEC-0103-ColBM, which lack the *parAB* and *psiAB* backbone also do not have a complete *tra* region, indicating that these two plasmids are likely not self-transmissible. In fact, pRSB107 and pAPEC-0103-ColBM were reported to be non-self-transmissible via conjugation (Szczepanowski et al., 2005; Johnson et al., 2010a). The extensive dissimilarities among the IncFIIA plasmids suggest that these plasmids are more diverse than previously thought.

Apart from being highly diverse, the multireplicon IncFIIA plasmids are also mosaic in nature (Figure 5; Supplementary Figure 2). This observation was supported by deviations in the GC content seen in certain regions of the plasmid (Supplementary Figure 2). Overall, the IncFIIA plasmids can be generally separated into five functional modules, with three conserved modules (regions encoding plasmid replication, maintenance and transfer) and two accessory regions (regions encoding antimicrobial resistance and virulence) being identified. These different modules can also be arranged in a non-orderly manner. Although the plasmid transfer module is conserved across all 18 studied plasmids, the genetic content within this region was found to be variable with truncations within the *tra* regions observed in several plasmids such as pRSB107, pS286ColV, and pO83-CORR (Figure 5; Supplementary Figure 2). More interestingly, the resistance regions of the plasmids are extremely diverse that none of the plasmids shared identical resistotypes (Figure 5) except for the highly related plasmids (such as pCS0010A and pCVM29188_146) that were isolated from the same source type (Supplementary Figure 2).

Variations can also be seen in closely related non-ColV/ColBM plasmids pRSB225 and pRSB107. Both pRSB225 and pRSB107 were obtained from unculturable bacteria from the same source and belonged to the same replicon type (F1:A1:B1). However, pRSB225 carried an additional iron acquisition system and a complete transfer region that were absent in pRSB107, suggesting that pRSB225 may have evolved with added adaptive traits. Likewise, an inversion of the DNA segment containing *sitABCD* and *iut/iucABCD* encoding iron acquisition systems was also observed for two highly similar ColV-plasmids pECOS88 and pCS0010A, which belonged to multireplicon type F24:A-:B1. An additional region encoding several resistance determinants is also found downstream of the F transfer region of pCS0010A but is completely absent in pECOS88 (Supplementary Figure 2). The dissimilarities observed between the closely related plasmids further substantiated the extensive diversity of IncFII plasmids.

### Phylogenetic analysis using FIIA replicon *repA1* and *tra* genes

Phylogenetic analysis using the single essential core gene of multireplicon IncFIIA plasmids, i.e., the *repA1* gene, revealed two distinct lineages, with all non-ColV/ColBM-producing resistance plasmids grouped closely in cluster A and all the ColV/ColBM-producing plasmids in Cluster B (Figure 4). Overall, the FAB formula for all studied plasmids showed concordant clustering with the phylogenetic tree generated using the RepFIIA *repA1* gene except for a ColV-producing plasmid (pAPEC-O2-ColV). Phylogenetic analysis was also carried out using 6 *tra* genes (*traI, traS, traT, traM, traJ*, and *traY*) which have been used to determine the phylogenetic relationship for plasmids in previous studies (Garcillán-Barcia et al., 2009; Peigne et al., 2009). It is noted that five out of the 18 IncFIIA plasmids do not harbor the 6 *tra* genes due to truncation in their respective *tra* regions, a phenomenon that is common for non-self-transmissible plasmids which was observed in our study (Figures 4, 5; Supplementary Figure 2). Based on the phylogenetic trees, the clustering of multireplicon IncFIIA plasmids using *tra* genes differed slightly from that of *repA1*, in which some ColV/ColBM plasmids were grouped together with non-ColV/ColBM plasmids and vice versa (Supplementary Figure 3). Due to some incongruence on the phylogenies inferred using *repA1* and *tra* genes, the core regions of the 18 plasmids were further determined using REALPHY. The phylogenetic tree built based on the core regions which encompass replication-related genes as well as conserved intergenic regions further substantiated the phylogeny inferred using only the *repA1* gene, where non-ColV/ColBM- and ColV/ColBM- producing plasmids are indeed separated into two distinct lineages (Supplementary Figure 4).

The findings where different *tra* genes showed varied evolutionary relationships also concurred with previous studies (Johnson and Nolan, 2009; Peigne et al., 2009). Peigne et al. (2009) reported that phylogenies inferred based on different *tra* genes can have conflicting topologies, probably due to the mosaic structure of the studied plasmids. In fact, our study showed the the presence of IS*66* along with deviations in the GC content within the plasmid transfer-encoding region (Figure 1), indicating that different regions of the plasmid may have been acquired independently from different sources, plausibly mediated by mobile genetic elements in the course of evolution. The *repA1* gene which is conserved across all studied plasmids also indicates its suitability to infer plasmid phylogeny, in agreement with previous studies (Sen et al., 2013; Shintani et al., 2015).

### pEC302/04-like plasmids are found in different environments

Of the 18 plasmids included in this comparative study, 17 were associated with strains from various extraintestinal sites, namely the ExPEC/uropathogenic *E. coli* (UPEC), ExPEC/neonatal meningitis *E. coli* (NMEC), ExPEC/avian pathogenic *E. coli* (APEC), waste water treatment plant (WWTP) and avian *Salmonella enterica*. Detailed comparative analysis of these 18 IncFII plasmids revealed that pEC302/04 shared high synteny with regards to the addiction systems and plasmid transfer-encoding regions with pEC_L8 and pEFC36a. pEC_L8 is a virulent UPEC-associated plasmid that harbors one of the most widely disseminated resistance gene (CTX-M-15) globally (Smet et al., 2010), while pEFC36a was obtained from an unculturable bacterium from a waste water treatment plant (Rahube et al., 2014). These three plasmids, i.e., pEC302/04, pEC_L8 and pEFC36a, are clearly closely related as inferred from the phylogenetic trees based on core regions and all 6 *tra* genes despite their diverse backgrounds. This also indicates that pEC302/04-like plasmids are not restricted to the ExPEC host isolated from the tracheal aspirate.

### Typical features of ColV/ColBM and non-ColV/ColBM plasmids

ColV and ColBM plasmids are among the typical examples of ExPEC plasmids and are known to be highly virulent (Johnson and Nolan, 2009). Besides possessing the usual core region of IncFIIA plasmids, namely the *repA1, parAB* and *psiAB* genes, the ColV/ColBM plasmids also have an additional backbone region, the RepFIB (Figure 5). The RepFIB in ColV/ColBM plasmids is flanked by a number of virulence genes (Figure 5; Supplementary Figure 2). Outer membrane protease (OmpT), hemolysin (HlyF), ABC transport system (Ets), increasing serum sensitivity (Iss), and salmochelin siderophore (IroBCDEN) are core virulence determinants found downstream of RepFIB while the aerobactin siderophore (Iut/IucABCD) and Sit iron and manganese transporter (SitABCD) are the conserved virulence determinants located upstream of RepFIB. All the aforementioned virulence encoding genes together with *repFIB* constitute the “constant region” of typical ColV/ColBM plasmids, a finding that concurred with a previous report (Johnson and Nolan, 2009). On the other hand, the temperature-sensitive hemagglutinin (Tsh) and the novel transport system (Eit) forms the “variable” region of ColV/ColBM plasmids (Figure 5; Supplementary Figure 2).

All non-ColV/ColBM plasmids that were analyzed in this work contained a region encoding multidrug resistance which is found proximal to the *repA1* gene. The non-ColV/ColBM resistance plasmids harbored different types of resistance genes that confer resistance to a wide range of antimicrobials with some of these being the first drug of choice for therapeutic treatment (Antibiotic Guidelines, 2015–2016).

Apart from resistance and virulence determinants, the ColV/BM- and non-ColV/ColBM-producing plasmids also harbor distinct subsets of TA systems. In general, *pemI/pemK, vagC/vagD*, and *ccdA/ccdB* were found to be more common in non-ColV/ColBM plasmids while *yacA/yacB* was more frequently found in ColV/ColBM plasmids (Figures 4, 5). To further support our observation, the genomes of 12 plasmid from ExPEC that were not included into this comparative study (non-closely related to pEC302/04) but have been well described in previous studies (Tivendale et al., 2004; Fricke et al., 2008; Woodford et al., 2009; Cusumano et al., 2010; Johnson et al., 2010b; Smet et al., 2010; Andersen et al., 2013; Brolund et al., 2013; Zong, 2013; Wijetunge et al., 2014; Phan et al., 2015) were further analyzed for the presence of different TA systems. The additional ColV/ColBM-producing plasmids incorporated in this further analysis may seem few (*n* = 4), but to the best of our knowledge, encompassed all ColV/ColBM-plasmids of IncF from ExPEC that have been published. The results obtained substantiated the fact that *yacA/yacB* appeared to be over represented in ColV/ColBM-producing plasmids. On the other hand, *vagC/vagD, ccdA/ccdB*, and *pemI/pemK* are highly common, but not limited to non-ColV/ColBM plasmids (Supplementary Table 4).

There are two possible explanations to describe such observation. Firstly, the association of certain TA systems with particular types of plasmids may confer selective fitness advantages to their bacterial hosts. Recently, Norton and Mulvey (2012) reported the association of a specific subset of chromosomally-encoded TA systems (i.e., YefM-YoeB, YbaJ-Hha, and PasT-PasI) with ExPEC isolates. These TA systems were able to confer increased resistance toward environmental stresses (such as nutrient limitation, oxidative and nitrosative stresses) by inducing persister cell formation. Some of these TA systems even contributed to bacterial pathogenesis by enhancing the colonization and survival of ExPEC isolates at human extraintestinal sites (Norton and Mulvey, 2012). Another possible interpretation for this observation is that a particular TA system such as *yacA/B* may have co-transferred together with the colicin V/BM encoding regions (purple regions in Figure 5) into the IncF plasmids during the course of evolution, as *yacA/yacB* genes are located in the vicinity of the colicin-encoding regions. Regardless of which underlying mechanism, the finding where certain TA system(s) are more commonly found in specific plasmid types indicate that TA systems may be useful for inferring phylogeny and evolutionary processes of selected bacterial plasmids.

### Uncommon plasmids that harbor both virulence and resistance determinants

Although, the 18 multireplicon IncFIIA plasmids included in this study were obtained from diverse sources, a number of genetic features are conserved within a specific type of plasmid (i.e., ColV/ColBM and non-ColV/ColBM plasmids). For instance, the MDR-encoding regions are frequently associated with non-ColV/ColBM resistance plasmids whereas the ColV/ColBM plasmids often carry virulence-encoding regions (Figures 4, 5, Supplementary Figure 2). However, plasmids carrying both features (MDR- and virulence-encoding regions) have been reported but at low occurrence (Johnson and Nolan, 2009).

Although, genes encoding for RepFIB, SitABCD, and/or aerobactin (IutA-IucABCD) are typically found in the “constant” virulence regions of ColV/ColBM plasmids, these regions have also been identified in some non-ColV/ColBM plasmids (underlined in black in Figure 5; Supplementary Figure 2). In our findings, pEC302/04 harbors RepFIB with iron acquisition systems but is lacking in other “constant” ColV/ColBM-plasmid virulence regions downstream of RepFIB. Similarly, such unusual occurrence has been described earlier in non-ColV/ColBM plasmids pRSB225 and pRSB107, which were believed to be the evolutionary intermediates of ColV/ColBM plasmids (Johnson and Nolan, 2009).

Another unusual genetic combination for the multireplicon IncFIIA plasmids was observed for ColV/ColBM plasmids pO83-CORR and pAPEC-O103-ColBM. Besides carrying the “constant” and “variable” virulence encoding region of ColV/ColBM-plasmids, pO83-CORR and pAPEC-O103-ColBM also harbor a multidrug resistance (MDR)-encoding region (Figure 5), and hence were termed as hybrid plasmids (Johnson et al., 2010a). Such phenomenon has also been reported for an atypical ColV/ColBM plasmid pSMS35_130 (Fricke et al., 2008; Johnson and Nolan, 2009). Remarkably, the constant “virulence” region of ColV/ColBM of all three hybrid plasmids (pO83-CORR, pAPEC-O103-ColBM, and pSMS35_130) are incomplete, where Ets and OmpT (which are the conserved virulence determinants of ColV/ColBM plasmids) were absent in pO83-CORR and pAPEC-O103-ColBM, respectively.

Interestingly, we found that the MDR-encoding regions for all three hybrid plasmids pO83-CORR, pAPEC-O103-ColBM, and pSMS35_130 were located in close proximity and downstream of the RepFIIA and PemI/PemK TA system, a genetic content and order that were previously found in non-ColV/ColBM resistance plasmids (Figure 5; Supplementary Figure 2; Johnson and Nolan, 2009). Nevertheless, ColV plasmids pCVM29188_146 and pCS0010A that contained the complete “constant” virulence region of ColV/ColBM plasmids also harbored antibiotic resistance genes, but at an atypical site, downstream of the F transfer region (Figure 5; Supplementary Figure 2). Although, the types of resistance genes carried by pCVM29188_146 and pCS0010A were similar (i.e., *strA, strB*, and *tet*) to non-ColV/ColBM plasmids, the number of resistance genes were relatively fewer when compared to the MDR-encoding regions located proximal to *pemI*/*pemK*. In contrast, none of the typical ColV/ColBM plasmids that carried the complete “constant” virulence region harbored MDR-encoding regions that were located proximal to the *repA1* and *pemI/K* genes (Figure 5; Supplementary Figure 2). Collectively, gene loss is observed in either MDR- or virulence- encoding regions of all ColV/ColBM hybrid plasmids (pO83-CORR, pAPEC-O103-ColBM, pSMS35_130, pCVM29188_146, and pCS0010A; Szczepanowski et al., 2005; Fricke et al., 2008; Johnson and Nolan, 2009; Johnson et al., 2010a; Wibberg et al., 2013). Although, the evolutionary forces contributing to the genetic makeup of these plasmids are not completely understood, we speculate that gene loss may be adaptive and beneficial to the bacterial host due likely from the reduced genetic burden. Hence, none of the ColV/BM-producing plasmids that harbored complete core set of virulence repertoire carried the MDR-encoding regions at the usual integration sites.

## Conclusion

The presence of both virulence and resistance determinants in the ExPEC IncFIIA plasmids is of public health importance as bacterial hosts harboring these plasmids can evade antimicrobial therapy, leading to ineffective infection control. Such situation is especially worrisome when these plasmids are transmissible, a phenomenon which is common among the plasmids analyzed in this work. Our study revealed that pEC302/04-like plasmids were found in diverse environments and strains harboring these plasmids were obtained at various extraintestinal sites (including urinary and respiratory tracts). The detailed comparative sequence analysis of pEC302/04 and 17 other IncFII plasmids from various sources showed that *repA1* is the only conserved gene, indicating the extensive diversity of these plasmids. Phylogenetic analysis using *repA1* enabled the separation of the ColV/ColBM- and non-ColV/ColBM-producing plasmids into two distinct groups. A number of multireplicon IncFIIA ExPEC plasmids that possess an atypical combination of genetic materials which include both virulence and resistance determinants were also highlighted. Interestingly, a “reduced” set of virulence and resistance determinants were found in the hybrid plasmids and possible evolutionary intermediates of ColV/ColBM plasmids, indicating that the carriage of both fitness traits in a single plasmid may incur a fitness cost to the bacterial host. Notably, specific TA systems were more commonly found in certain types of ExPEC plasmids, which hints at possible interesting relationships between TA systems and ExPEC pathogenesis.

## Author contributions

WSH designed the study, analyzed and wrote the manuscript. WSH and KPY interpreted the data. WSH, KPY, CCY, KLT helped in drafting and critically reviewed the manuscript and contributed important intellectual output. KLT provided funding for the project. CCY and KLT supervised the project. All authors contributed to the editing of the manuscript and all authors read and approved the final manuscript.

## Funding

We thank the University of Malaya for facilities and support. This work was supported by High Impact Research Grant-Molecular Genetics (reference UM.C/625/1HIR/MOHE/-02 [A000002-5000]) from University of Malaya and MOSTI (GA013-2013). WSH is supported by full time research assistantship from HIR of University of Malaya.

### Conflict of interest statement

The authors declare that the research was conducted in the absence of any commercial or financial relationships that could be construed as a potential conflict of interest.

## Supplementary material

Supplementary Figure 1 **Circular view showing the (A) core genome and (B) accessory genomes of 18 IncF plasmids**. The circular view was constructed using Gview with plasmid pEC302/04 assigned as the seed genome.

Supplementary Figure 2 **Unannotated pairwise nucleotide comparative map of 7 selected multireplicon IncFIIA plasmids**. Same-strand DNA similarity is shaded light blue while reverse similarity is shaded light yellow. Coding sequences are displayed as rectangles. Major features are displayed in colors based on functional annotations: orange, replication; pink, plasmid transfer region; yellow, addictive systems; red, iron acquisition systems; purple, colicin V/BM; blue, antimicrobial resistance; green, other virulence factors; white, other genes. GC content of selected plasmids are shown above their respective genetic maps, with red and blue region showing GC content above and below 50%, respectively.

Supplementary Figure 3 **Phylogenetic trees of *tra* genes for 18 multireplicon IncFIIA plasmids. (A)**, *tra*I; **(B)**, *tra*T; **(C)**, *tra*M; **(D)**, *tra*Y; **(E)**, *tra*S; **(F)**, *tra*J. Maximum likelihood (ML) method under the General Time Reversible plus Gamma model was used to construct the phylogenetic trees using MEGA5 supported with bootstrapping (1000 replicates). Bootstrap value with percentages equals or greater than 50% were shown on branches and value less than 50% have been collapsed. The 6 *tra* genes of plasmids pEK499, pRSB107, pAPEC-O1, pS286ColV, pAPEC-O103-ColBM were not included due to truncation in their respective *tra* regions.

Supplementary Figure 4 **Comparison of phylogenetic trees constructed for 18 multireplicon IncFIIA plasmids using (A), *repA1*; (B), core regions determined using REALPHY**. Maximum likelihood (ML) method under the General Time Reversible plus Gamma model was used to construct the phylogenetic trees using MEGA5 supported with bootstrapping (1000 replicates).

Supplementary Table 1 **Primers used for plasmid pEC302/04 gap closing**.

Supplementary Table 2 **Iron acquisition systems**.

Supplementary Table 3 **Annotation for plasmid pEC302/04 using RAST**.

Supplementary Table 4 **Presence and absence of TA systems in the plasmid sequences**.
