# Supplementary figures and images for: The Complete Sequence and Comparative Analysis of a Multidrug-Resistance and Virulence Multireplicon IncFII Plasmid pEC302/04 from an Extraintestinal Pathogenic Escherichia coli EC302/04 Indicate Extensive Diversity of IncFII Plasmids
Source: Front Microbiol. 2016 Jan 11;6:1547. doi: 10.3389/fmicb.2015.01547 (PMC4707298; doi:10.3389/fmicb.2015.01547)

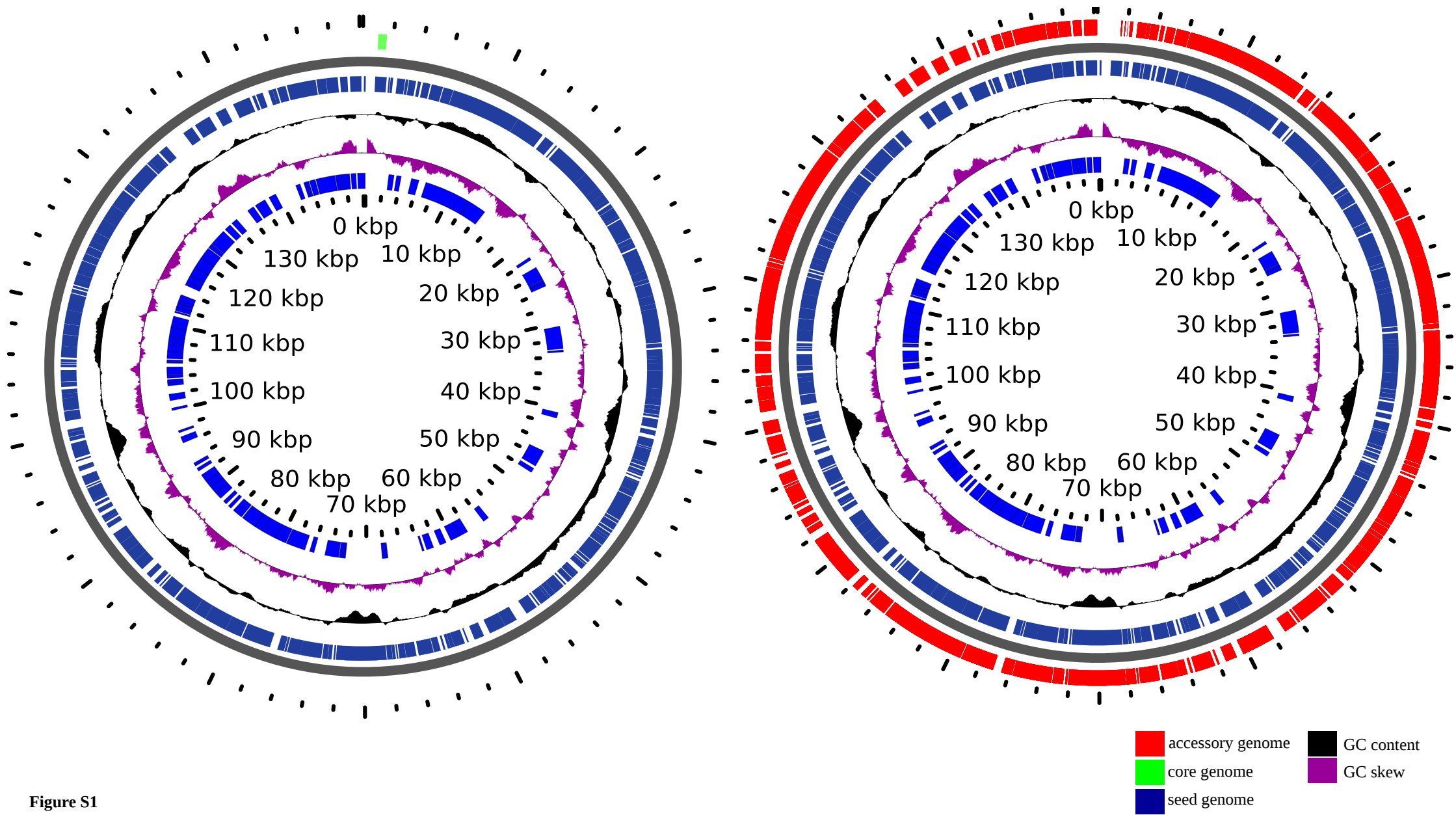

Figure S1

Supplement: Supplementary Figure 1 — Circular view showing the (A) core genome and (B) accessory genomes of 18 IncF plasmids. The circular view was constructed using Gview with plasmid pEC302/04 assigned as the seed genome. [file Image1.PDF]

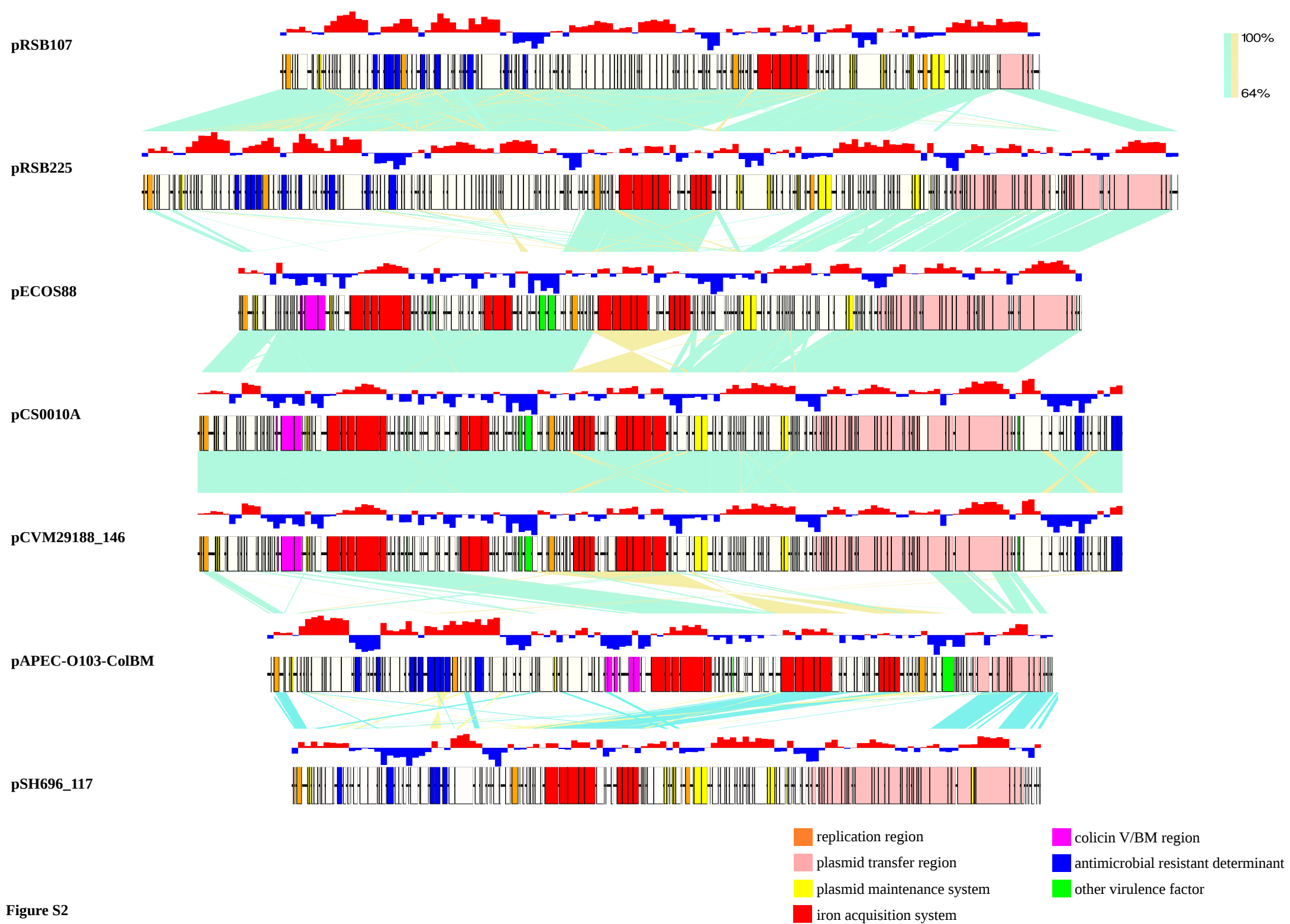

Supplement: Supplementary Figure 2 — Unannotated pairwise nucleotide comparative map of 7 selected multireplicon IncFIIA plasmids. Same-strand DNA similarity is shaded light blue while reverse similarity is shaded light yellow. Coding sequences are displayed as rectangles. Major features are displayed in colors based on functional annotations: orange, replication; pink, plasmid transfer region; yellow, addictive systems; red, iron acquisition systems; purple, colicin V/BM; blue, antimicrobial resistance; green, other virulence factors; white, other genes. GC content of selected plasmids are shown above their respective genetic maps, with red and blue region showing GC content above and below 50%, respectively. [file Image2.PDF]

(a) traI

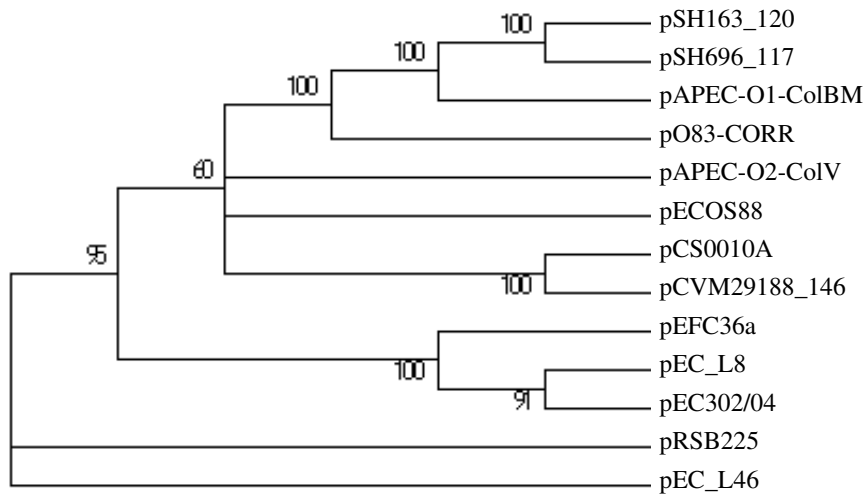

(d) traY

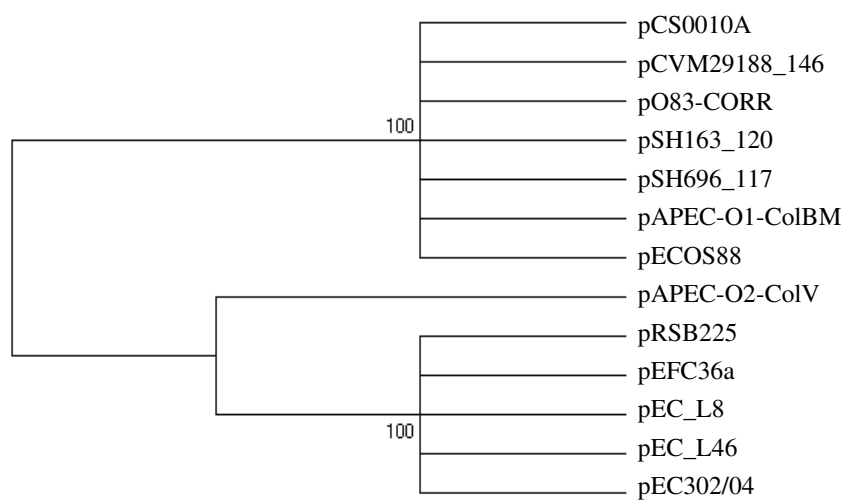

(b) traT

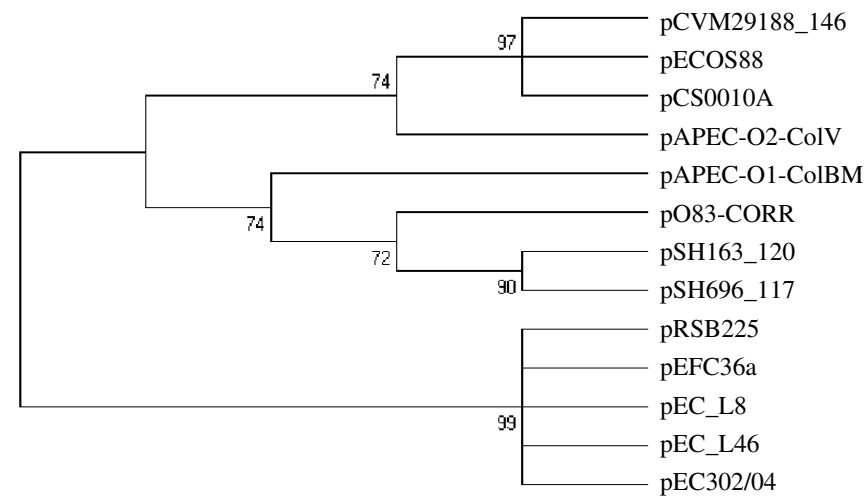

(e) traF

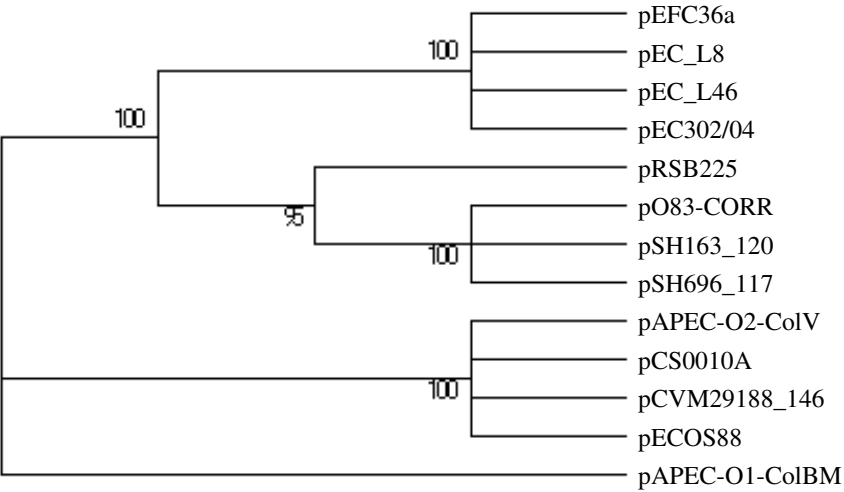

(c) traM

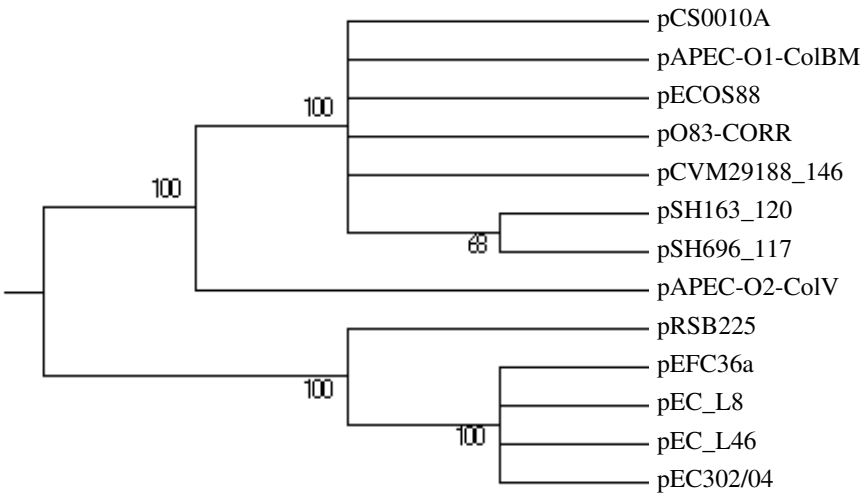

(e) traJ

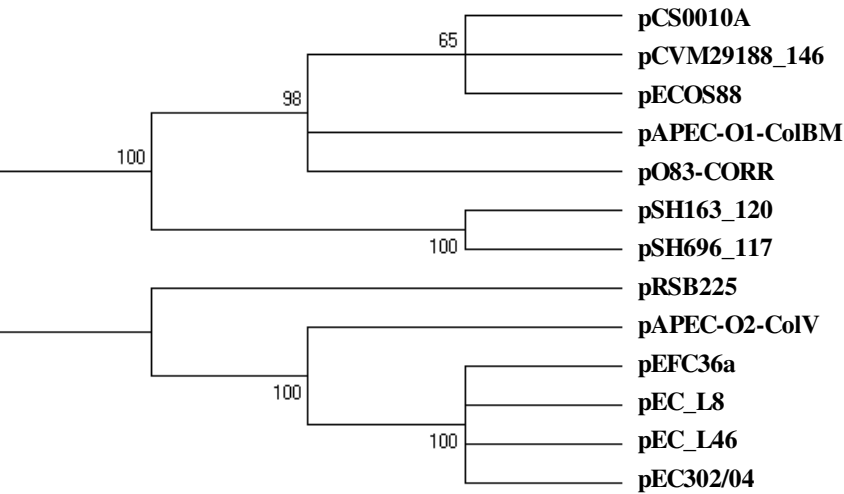

Figure S3

Supplement: Supplementary Figure 3 — Phylogenetic trees of tra genes for 18 multireplicon IncFIIA plasmids. (A), traI; (B), traT; (C), traM; (D), traY; (E), traS; (F), traJ. Maximum likelihood (ML) method under the General Time Reversible plus Gamma model was used to construct the phylogenetic trees using MEGA5 supported with bootstrapping (1000 replicates). Bootstrap value with percentages equals or greater than 50% were shown on branches and value less than 50% have been collapsed. The 6 tra genes of plasmids pEK499, pRSB107, pAPEC-O1, pS286ColV, pAPEC-O103-ColBM were not included due to truncation in their respective tra regions. [file Image3.PDF]

(a)

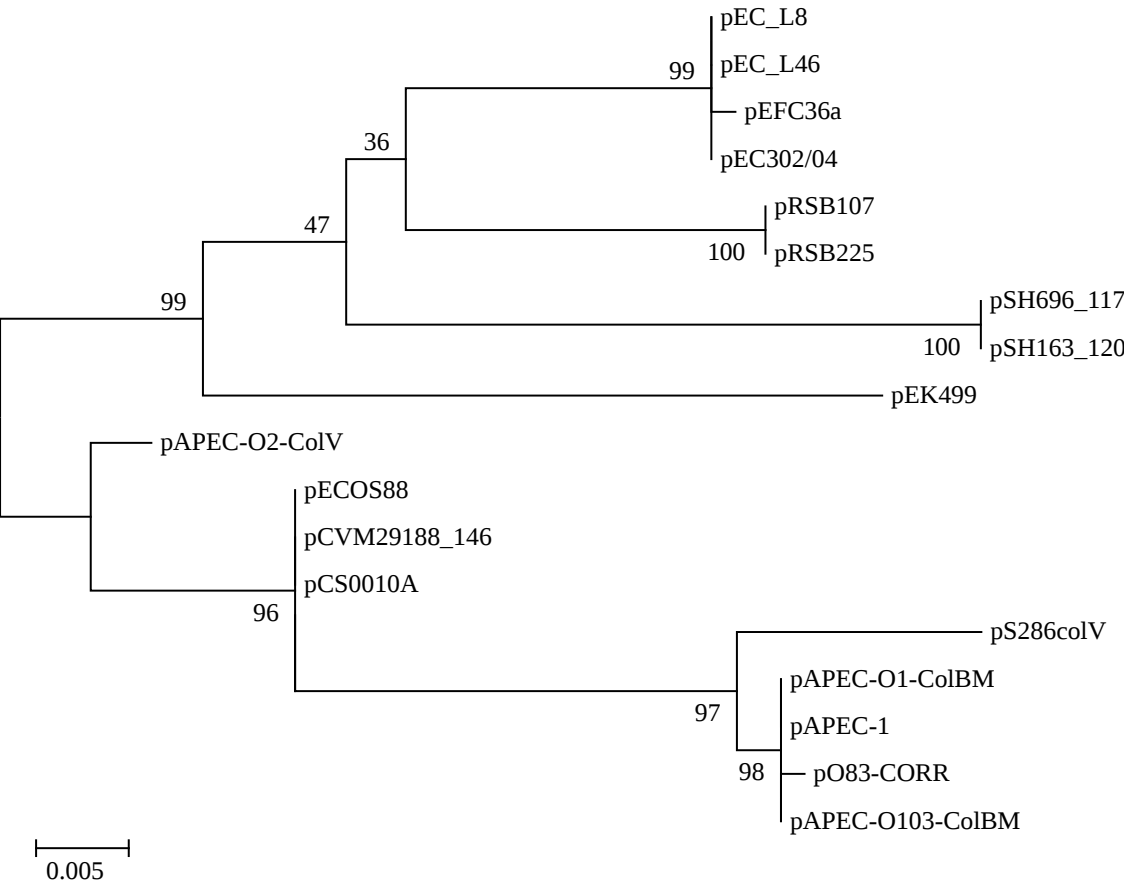

(b)

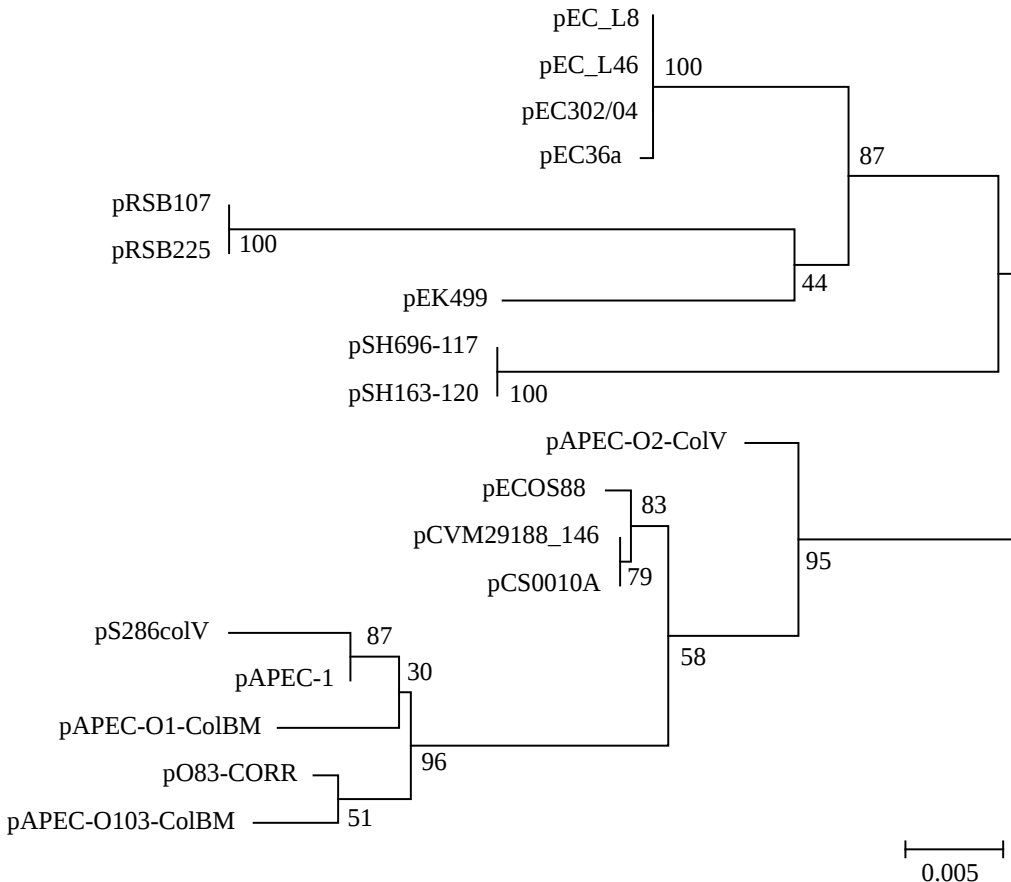

Figure S4

Supplement: Supplementary Figure 4 — Comparison of phylogenetic trees constructed for 18 multireplicon IncFIIA plasmids using (A), repA1; (B), core regions determined using REALPHY. Maximum likelihood (ML) method under the General Time Reversible plus Gamma model was used to construct the phylogenetic trees using MEGA5 supported with bootstrapping (1000 replicates). [file Image4.PDF]
